# 

**Published:** 1893-09-02

**Authors:** 


### CONTENTS.

**The Mystery of Influenza** - - - 353

**Around the Hospitals** - - - 354

**Diet in Disease.** By Mrs. ERNEST HART. XXIX.—Chronic Bright's Disease (*continued*) - - - 355

**Gleanings in Science and Medicine** - - - 356

**Women's Work in South Africa** - - - 357

**The Hospital Clinic**—

THE TREATMENT OF TUBERCULAR ENTERITIS - - - 361

THE LONDON HOSPITAL.—The Treatment of some Forms of Chronic Ulcer - - - 361

NEW DRUGS AND PREPARATIONS. Analgen - - - 363

NEW APPLIANCES AND THINGS MEDICAL—New Preparations from the Sanitas Company - - - 364

**Famous Poisoners in Fiction.** IV.—A Royal Poisoner - - 358

**Annotations.**—Parents and Schoolboys' Loyalty—A Nation of Tea-Drinkers—Leprosy in Trinidad - - - 359

**Notes and News** - - - 360

**The Institutional Workshop**—

HOSPITAL CONSTRUCTION.—The Sisters' Hospital, St. Alban's - 365

THE STORY OF THE INSANE FROM YEAR TO YEAR.—The Care of the Feeble-minded. I.—In Childhood - - - 366

PRACTICAL DEPARTMENTS.—Sponges. CONSTRUCTION NOTES - 367

**Editor's Letter-Box.**—Practical Experience in Poultice Making - 368

**Scraps and Gleanings** - - - 368

**The Book World of Medicine and Science** - - - vii

**Medical Vacancies and Appointments** - - - ix

### EXTRA SUPPLEMENT.—THE NURSING MIRROR.

**News from the Nursing World.**—Our Christmas Competitions—The Nightingale Fund—Miss Victoria Jones—Maternity Nurses—Village Nurses—"The Hospital" Competition for Nurses' Homes—Careless Correspondents—An Ambitious Cottage Hospital—A Proof of Usefulness—A Hero from a Convalescent Home—Nursing at Dundee—Bristol Royal Infirmary — Short Items - - - ccxxi
**Practical Information Column.**—The Parish Nurse - ccxxiii
**The Benefits of Alumnæ Associations** By Miss McIsaac - - - ccxxiv
**"The Truth About the London Hospital"** - - ccxxv
**The World of Nurses.** As Seen by an Unprofessional Correspondent. III.—Nurses and Dress - - - ccxxv
**Everybody's Opinion.**—Nurses' Uniforms—A Home for Incurables - - - ccxxvi
**Novelties for Nurses.** — Woollen Underclothing — "The Leicester" Accouchement Binder - - - ccxxvi
**Death in Our Ranks.**—**Minor Appointments** - - ccxxvii
**Notes and Queries.**—**For Reading to the Sick.**—Prayer ccxxvii
**The Muses' Looking Glass.**—The Lesson Learnt - - ccxxviii
**Australian Notes.** From Our Special Correspondent - - ccxxviii
**The Book World for Women and Nurses** - - - ccxxix

